# Vulnerability of Venezuelan immigrants living in Boa Vista, Roraima*

**DOI:** 10.1590/1980-220X-REEUSP-2023-0074en

**Published:** 2023-09-15

**Authors:** Aristides Sampaio Cavalcante, Maria Amélia de Campos Oliveira, Emiko Yoshikawa Egry

**Affiliations:** 1 Instituto Federal de Roraima, Departamento de Graduação, Boa Vista, RR, Brazil.; 2 Universidade de São Paulo, Escola de Enfermagem, Departamento de Saúde Coletiva, São Paulo, SP, Brazil.

**Keywords:** Disaster Vulnerability, Nursing, Emigrants and Immigrants, Public health, Health policies, Vulnerabilidad ante Desastres, Emigrantes e Inmigrantes, Salud Pública, Políticas de Salud, Vulnerabilidade a Desastres, Enfermagem, Emigrantes e Imigrantes, Saúde Pública, Políticas de Saúde

## Abstract

**Objective:**

To identify the social and health vulnerabilities of Venezuelan immigrants living in Boa Vista, Roraima.

**Method:**

Mixed methods research, with concomitant transformative strategy. In the quantitative phase, analysis of management documents for the state of Roraima and the capital Boa Vista were carried out. In the qualitative, open interviews with 16 Venezuelan immigrants, 14 nursing professionals and 8 public managers and a focus group with 12 nursing professionals. The webQDA software was used to organize the data and the content and discourse analyzes were based on Bardin and on dialectic-hermeneutics, according to Minayo.

**Results:**

The main vulnerabilities identified were the absence of public policies for immigrants, the fragility of links with health services and language differences.

**Conclusion:**

The vulnerabilities of Venezuelan immigrants are linked to their ways of living and working. Health institutions and organizations are not fully qualified to serve them. Specific policies are needed for this social group and to qualify institutions and health professionals to implement these policies.

## RESUMO

**Objetivo:**

Identificar as vulnerabilidades sociais e de saúde de imigrantes venezuelanos residentes em Boa Vista, Roraima.

**Método:**

Pesquisa de métodos mistos, com estratégia transformativa concomitante. Na fase quantitativa, foram realizadas análises de documentos de gestão do estado de Roraima e da capital Boa Vista. Na qualitativa, entrevistas abertas com 16 imigrantes venezuelanos, 14 profissionais de enfermagem e 8 gestores públicos e um grupo focal com 12 profissionais de enfermagem. Utilizou-se o *software* webQDA para organização dos dados, e as análises de conteúdo e de discurso foram baseadas em Bardin e na hermenêutica-dialética, segundo Minayo.

**Resultados:**

As principais vulnerabilidades identificadas foram a ausência de políticas públicas para imigrantes, a fragilidade dos vínculos com os serviços de saúde e as diferenças de idioma.

**Conclusão:**

As vulnerabilidades dos imigrantes venezuelanos encontram-se vinculadas aos seus modos de viver e de trabalhar. As instituições e organizações de saúde não se encontram plenamente qualificadas para atendê-los. São necessárias políticas específicas para esse grupo social e qualificar instituições e profissionais de saúde para execução dessas políticas.

## RESUMEN

**Objetivo:**

Identificar las vulnerabilidades sociales y sanitarias de inmigrantes venezolanos residentes en Boa Vista, Roraima.

**Método:**

Es una investigación de métodos mixtos, con estrategia transformativa concomitante. En la fase cuantitativa se llevó a cabo el análisis de documentos de gestión del estado de Roraima y de su capital, Boa Vista. En la fase cualitativa, se realizaron entrevistas abiertas con 16 inmigrantes venezolanos, 14 enfermeros y 8 gestores públicos y un grupo focal con 12 profesionales de enfermería. Se utilizó el *software* webQDA para organizar los datos y el análisis de contenido y discurso estuvo basado en Bardin y en la hermenéutica-dialéctica, según Minayo.

**Resultados:**

Las principales vulnerabilidades fueron la falta de políticas públicas hacia los inmigrantes, la fragilidad de los vínculos con los servicios sanitarios y las diferencias lingüísticas.

**Conclusión:**

Las vulnerabilidades de los inmigrantes venezolanos están relacionadas con su forma de vida y trabajo. Las instituciones y organizaciones sanitarias no tienen suficiente capacidad para atenderlos. Se necesitan políticas específicas para este grupo social y preparación de las instituciones y profesionales de la salud para implementar dichas políticas.

## INTRODUCTION

Immigrants constitute a vulnerable population, especially in countries with emerging economies^(1)^. Venezuelan immigrants who have moved to Brazil since 2015 have numerous social vulnerabilities. After surviving the shortage of basic consumer goods in their homeland and crossing the border, exposing themselves to the risk of death on drug trafficking routes, they arrive in Boa Vista, capital of the state of Roraima, where they suffer multiple prejudices arising from xenophobia, which results in social exclusion^(2)^.

National and international studies corroborate the importance of investing in research on the social and health vulnerabilities of immigrants^(3,4)^, people who, impacted by their displacements, often forced, have a high potential for the development of diseases and injuries of both physical and emotional nature^(4)^. Considering mental health issues, suicides, anxiety disorders and depression stand out as the main mental health problems that affect this population^(5)^.

Illnesses and physical injuries result, for the most part, from poor living and working conditions. Upon arriving in a new country, without any documentation proving that their stay there is legal, immigrants have their labor exploited and are often forced to work long hours and without decent remuneration, even subjected to conditions analogous to slavery^(6)^. Something similar has been observed in the reality of Venezuelan immigrants who settle in Roraima, specifically in the capital Boa Vista.

The national press, as well as organizations for the protection of immigrants and refugees, have identified several episodes of flagrant disrespect for the human rights of Venezuelans in Boa Vista and have been warning of the need to intensify efforts to face these situations^(7)^. Identifying the health vulnerabilities experienced by Venezuelan immigrants and acting to face them contributes to the achievement of citizenship and consequent improvement in the quality of life of these people^(8)^. Thus, the objective of the present study was to identify the health vulnerabilities of Venezuelan immigrants residing in Boa Vista.

## METHOD

### Study Design

Mixed methods research, with an exploratory, analytical and descriptive character, which used the concomitant transformative strategy. The Concomitant Transformative strategy is guided by the researcher from a specific theoretical perspective and collects both quantitative and qualitative data^(9)^.

### Population

The study included 16 Venezuelan immigrants, 26 professionals (14 interviewees and 12 participants in a focus group) and 8 public managers who work in health centers in the capital Boa Vista, at the three levels of care.

### Data Collection and Analysis

Data collection took place between July and October 2021, in two phases: in the quantitative phase, information was collected from state and municipal management reports for the years 2018, 2019 and 2020, from municipal and state health surveillance, policies and public actions built and executed in Roraima to compose the local epidemiological profile and identify demands related to Venezuelan immigrants, particularly the most vulnerable. In the qualitative phase, it was decided to carry out a focus group with 12 health professionals belonging to the Nursing teams of different health centers in the capital, which allowed them to express their opinions about the care given to Venezuelan immigrants in their respective health centers. The open interviews, on the other hand, aimed to know the individual perspectives on the studied phenomenon and were carried out with 14 other nursing professionals, 08 managers and 16 Venezuelan participants.

The use of open interviews is justified by the need to freely conduct the collection of information, as it allows the conversation to “float” on any subject that is deemed appropriate or necessary^(10)^. Due to cultural and language differences, we had the support of a cultural intermediary of Venezuelan origin. The number of interview participants was defined by saturation^(11)^. Thus, the importance of the participants in the studied phenomenon was taken into account; the characteristics, experiences and expressions that supported the participants’ speeches; sociocultural, political, economic, professional, health issues and other aspects related to the subjectivity of the groups involved. The decision to end the interviews was taken for each group, after identifying repetitions, similarities and absence of complementary statements.

The interviews and the focus group were audio recorded and later transcribed. The audios in Spanish were transcribed, translated and checked with the help of the cultural intermediary. Data analysis was supported by the webQDA software to support qualitative data analysis^(12)^. Bardin’s^(13)^ content analysis and Minayo’s^(11)^ hermeneutic-dialectical analysis were used to analyze the discourses emerging from the interviews and the focus group.

The theoretical framework that guided the discussion of the findings was the social determination of the health-disease process which, according to Breilh^(14)^, considers the mode of production and social reproduction as a determinant of the epidemiological profile of a population.

### Methodological Strategies

**Chart 1:**
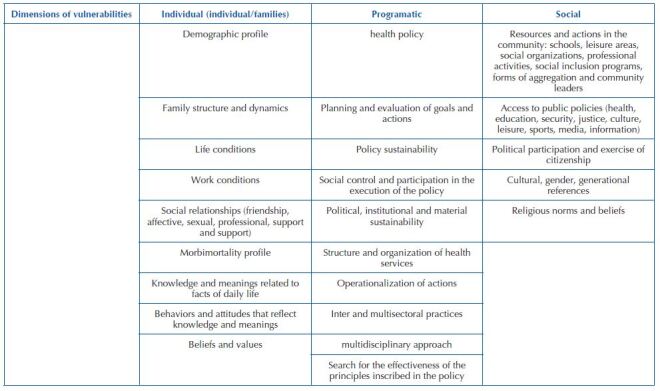
Vulnerability markers related to diseases and injuries in each territory – Boa Vista, RR, Brazil, 2023.

**Source:** Evaluative perspectives of health vulnerabilities. In: Health needs from the perspective of Primary Care: a guide for researchers.

Chart 1 ^(15)^, presents the markers of vulnerability to diseases and injuries in a given territory, used in this study.

### Ethical Aspects

The research was submitted, approved on March 24, 2021, by the Research Ethics Committee of the USP School of Nursing, under opinion No. 4,608,934, in compliance with Resolution No. 466 of December 12, 2012, which determines standards and regulatory guidelines in research involving human subjects. The Informed Consent Form (TCLE) was issued in the native languages of the study participants.

## RESULTS

The vulnerabilities of the Venezuelan population are presented below, extracted from the analysis of management documents, the lines produced in the interviews, as well as the focus group and which were organized according to vulnerability markers^(15)^, in the individual, programmatic and social dimensions.

### Individual Dimension

#### Demographic profile:

57% of the Venezuelan immigrant population is male, young adults, aged between 20 and 39 years. Schooling is low and 37% do not have the level that, in Brazil, would correspond to complete primary education. The average income of 71% of Venezuelans in Boa Vista does not exceed one and a half minimum wages, around US$193^(16)^. Since the reopening of the border in March 2021, most have arrived in Brazil from the Venezuelan states close to the border, such as Amazona, Bolívar, Monagas and Anzoátegui^(17)^. In the city of Boa Vista, they mainly occupy the neighborhoods on the outskirts of the west zone, known as the Red Zone, due to its high crime rates.

#### Family structure and dynamics:

Single, biparental and extended families were identified. Families with nine people were seen living in spaces that could fit, at most, three or four. Among the Venezuelan respondents, the families were patriarchal, with the exception of three single-parent families, in which the heads were women who lived with their sons, daughters and mothers;

#### Life conditions:

Immigrants do not enjoy good living conditions. The number of unemployed people in Boa Vista is high (about 70% of the local immigrant population). There is a lack of resources to buy food, difficulties in obtaining documents required for access to work, health services and social benefits;

#### Work conditions:

Immigrants occupy informal jobs or underemployment. These people represent about 25% of residents in Boa Vista and are subjected to unhealthy working conditions, with wages lower than those of Brazilians^(16)^;

#### Knowledge and meanings about daily life:

Immigrants strongly associate the feeling of being safe with socioeconomic conditions. Most respondents reported a sense of security for being in Brazil, given the financial achievements;

#### Social relationships:

Parties and get-togethers are common in the villas and residences where Venezuelans live, on the outskirts of Boa Vista. In the interviews, nursing professionals commented that the sexual and affective relationships of Venezuelans in Boa Vista are with multiple partners. According to reports from PHC and Maternity professionals, the use of condoms is not widely accepted among immigrants;

#### Morbimortality profile:

No studies were found that accurately described the morbidity and mortality profile of the Venezuelan immigrant population in Roraima, as it is a recent phenomenon. However, several studies identified in the preliminary scoping review^(18)^, as well as testimonials from managers and nursing professionals, signaling violence as one of the main causes of death among Venezuelans;

#### Behaviors and attitudes that reflect knowledge and meanings:

The main behaviors and attitudes relate to the fears that Venezuelan immigrants have of not being assisted or having their rights denied due to the lack of adequate documentation to guarantee their permanence in the country;

#### Beliefs and values:

No relevant situations or speeches were identified on this topic, however, when they expressed their satisfaction for having achieved a paid activity or for having achieved some socioeconomic stability, the expression “Thanks to God” was frequently repeated by the immigrants.

### Programmatic Dimension

#### Health policy:

No public health policies specifically aimed at the Venezuelan immigrant population were identified in Roraima, nor in the capital Boa Vista;

#### Planning and evaluation of goals and actions:

Considering the absence of public health policies, the only planning mentioned by the managers referred to the demographic growth of the local population, to organize care flows, coordinate PHC actions and adjust the receipt of resources by the upper levels of management, in this case, the municipality of Boa Vista and the government of the state of Roraima. The analysis of management documents did not identify significant actions with the immigrant population, in the state or in the capital;

#### Policy sustainability:

The actions of Operation Acolhida, together with other non-governmental and human rights protection organizations (United Nations High Commissioner for Refugees, International Organization for Migration and Adventist Development and Relief Agency) represent the scope of everything that is carried out in Roraima to meet the needs of the Venezuelan population there;

#### Social control and participation in the execution of the policy:

There is no evidence of effective participation by Venezuelan immigrants in any decision-making process, therefore, one cannot speak of social control on the part of a population that does not have representatives with the right to vocalize their social and health needs;

#### Political, institutional and material sustainability:

Polarized political positions were identified regarding the migratory crisis and its impacts in Roraima. On the one hand, the federal government and UN-affiliated institutions welcome, include and help refugees. On the other hand, the “neutrality” is observed in the lack of actions by the state government and the City Hall and the aversion to the presence of Venezuelans in the statements of local politicians that suggest the end of the Acolhida Operation in Roraima^(19)^;

#### Structure and organization of health services:

The professionals interviewed at the General Hospital indicate work overload, overcrowding, the essential need to hire more professionals and the inauguration of hospital annexes that were still under construction^(20)^. In PHC the situation is similar, however, there are more welcoming initiatives than in hospitals;

#### Operationalization of actions:

Actions to welcome immigrants are carried out by the Acolhida Operation in Pacaraima and Boa Vista. Actions by local public managers are scarce, punctual and depend on the initiatives of certain professionals or teams;

#### Inter and multisectoral practices:

The partnerships between the state government, the municipal government and the Acolhida Operation are topical. During 2018 and 2019, in a partnership between the state government and AMATUR Turismo, buses were made available to take immigrants who were no longer interested in staying in Brazil back to Santa Elena de Uairén^(21)^;

#### Multidisciplinary approach:

Actions with a multidisciplinary focus are carried out by partner organizations of the Acolhida Operation. Considering the absence of public health policies aimed at Venezuelans in Roraima, it was not possible to identify recognizably multidisciplinary practices in the health services and in the interviewees’ speeches;

#### Search for the effectiveness of the principles inscribed in the policy:

In the absence of specific public policies for Venezuelan immigrants in Roraima, the search for guaranteeing rights turns to the achievements of the New Migration Law. Affirmative actions developed by the United Nations High Commissioner for Refugees, International Organization for Migration, Adventist Development and Relief Agency, Federal University of Roraima, Federal Institute of Roraima and Diocese of Roraima provide training in several areas so that Venezuelans can receive certification and perform jobs with better pay.

### Social Dimension

#### Resources and actions in the community:

Venezuelan immigrants in Boa Vista do not have their own organization that voices their human rights through constituted leaders. They are inserted in the same sociocultural, political and economic matrix of Brazilians;

#### Access to public policies:

Relevant obstacles were identified in immigrants’ access to local public services, mainly health services, due to language barriers, geographic orientation and xenophobia. Venezuelans report waiting months for an exam or consultation with specialists, when they can find vacancies;

#### Political participation and exercise of citizenship:

Interviews with immigrants and analysis of management documents demonstrated the lack of social and political leadership of this population. More than that, the speech of some interviewees revealed how a segregated social system silences the voice of the refugee population;

#### Cultural, gender and generation references:

The interviewees expressed a deep sense of loss related to the separation from their country, their customs, their culture and, mainly, their families. Social gender stereotypes could be identified in the statements of the interviewees who claim to only get jobs as day laborers and cleaners and not being able to exercise their original professions. Nursing professionals mentioned that Venezuelan women establish sexual favoring relationships in exchange for housing and food;

#### Religious norms and beliefs:

Like the state, the municipality of Boa Vista has a population with 55% of people self-declared Catholics, followed by 33% Protestants, 10% Spiritualists and 2% belonging to other religions or who declare themselves agnostics^(22)^. In Venezuela, 85.7% of the population declare themselves Catholic, followed by 12% of Protestants and 2.3% of other religions^(23)^.

## DISCUSSION

The analysis of the results of this research showed the existence of multiple vulnerabilities that affect the Venezuelan immigrant population residing in Boa Vista, since their health needs are not being met. Economic neoliberalism, typical of the mode of production and social reproduction in Brazil and Roraima, centered on capital accumulation, is characterized by class subalternity, labor exploitation and prejudice against immigrants, and constitutes the main oppressive force that subordinates the Venezuelans^(24)^. Agribusiness and the illegal exploitation of gold move large sums and manage to co-opt immigrants in conditions of social vulnerability. Even under legal perspectives of work, immigrants are dispossessed, since local productive activities do not formalize employment relationships that would guarantee some form of social protection.

The specificities of the Venezuelan immigration phenomenon in Roraima present similarities with migration realities in other states of Brazil and in the world. In a scope review study on the vulnerabilities of Arab refugees residing in the city of São Paulo, Lima Jr et al.^(25)^ listed the main vulnerabilities identified in 40 articles that composed the review. Also in the study by Lima Jr et al.^(25)^, the elements of vulnerability highlighted the inequality and disadvantage of refugees in relation to the health system in the host countries. In the individual dimension of vulnerability, the incidence of psychological disorders stood out, whose susceptibility is ten times greater than other types of diseases or health problems. In the social dimension, the migratory phenomenon itself was cited as a condition of vulnerability for immigrants. In the programmatic dimension, the vulnerability markers that were highlighted were access to health services, the development of public health policies and health practices, consequences of weaknesses in the field of public policies aimed at the health of immigrants. The language barrier was the main barrier to accessing health services, followed by the waiting time for care and the cost of services.

With regard to laws and public policies to protect immigrants and refugees, Brazil has in its legal system the New Migration Law. Added to this is Law No. 13,684, of June 21, 2018, which served as a basis for the creation of the Acolhida Operation, in which a strong connection with the Universal Declaration of Human Rights and the principles and guidelines of the SUS can be identified. The analysis of data from management documents, interviews and focus groups shows that there are important inequalities experienced by immigrants, even with instruments of great potential to respond to health needs and combat their vulnerabilities in Brazil, especially in the states of the North region from Brazil, who are the most affected by the phenomenon of Venezuelan immigration.

As one of the most important vulnerabilities identified, the lack of public policies aimed at meeting the needs of Venezuelans constitutes a kind of “silence” that bothers those who recognize them as people with rights. The denial of assistance to immigrants by the government is related to an outdated protectionist logic, but which is still part of neoliberal policies in some European countries, as well as in the United States and more recently in Brazil^(26)^. Such policies are promoted and financed by conservative wings, for which immigrants and refugees represent potential threats to the rights of the native population and, consequently, to public order. Thus, they are not seen as subjects of law. Barriers imposed on immigrants reinforce racist, hierarchical and colonialist postures, while promoting the spread of xenophobia and exacerbating intolerance^(26)^.

The vulnerabilities of Venezuelan immigrants residing in Boa Vista are also determined by their working conditions. Different work environments produce different vulnerabilities, even for similar activities. In the logic of reproduction of social models, work processes can be protective or destructive. Social models are ways in which individuals interact within their particular dimensions and reproduce interaction formats determined by the prevailing economic macrostructure in a given location. This form of reproduction determines the most relevant aspects in people’s health process^(14)^.

Since survival is one of the main motivations for emigrating, Venezuelans in Roraima associate security with stable socioeconomic conditions. Such an understanding favors the perpetuation of the exploitation of these people’s labor, induces their entry into illegal activities, including mining and drug trafficking, which impacts their health profile. That is, Venezuelan immigrants engage in destructive work processes, occupy jobs with high risk, low pay and greater exposure to harm as a result of a process socially imposed by their work activities^(2)^.

The above assertions are corroborated by the data brought in the quantitative data collection section, which report an increase in the incidence rates of arboviruses (yellow fever, dengue, zika and chikungunya), malaria and leishmaniasis (resulting from the mining activity that occurs within the regions dense forest) violent deaths (homicides and traffic accidents), violence and sexual exploitation of Venezuelan women. Added to this are the high rates of respiratory diseases, parasites and diarrheal diseases that affect the child population and the increase in teenage pregnancy rates and complications typical of the gestational period^(27)^.

In addition to the living and working conditions, the condition of being a poor foreigner fosters xenophobia, which also leads to the segregation and marginalization of Venezuelans in the physical space of the municipality of Boa Vista, which is evidenced by the geographic layout of their places of residence^(28)^. Except for those who live in the 13 de Setembro and São Vicente neighborhoods, located around the Rondon shelters, all the other interviewees and their families lived in the west region of the capital, also known as the “Red Zone”, where rent values are cheaper. This is due to a series of facts, among which the high levels of criminality and the lack of public safety stand out. There are also settlements and occupations through invasions. Residents claim that the main motivation for their stay in these neighborhoods stems from public policies to promote settlements, something that has also become an objective for Venezuelans residing in the area^(29)^.

Another serious social and health problem that makes up the vulnerabilities of the immigrant population in Boa Vista is violence against Venezuelan women. In the Venezuelan ghettos of Boa Vista, sexual practices with multiple partners, both men and women, reported by nursing professionals and managers, may have their origin in social confinement^(28)^ which is characterized as a socio-organizing device that imprints on the immigrant a violence constituted from the isolation and marginalization in physical spaces of a certain place. The option not to use condoms may have the same motivation or, from a gender perspective, derive from female subalternity imposed by the sexist social model^(30)^. Even if the present study does not have enough elements to elucidate such motivations, the fact is that STI rates among Venezuelans are quite high^(27)^.

In tackling health vulnerabilities, ensuring quality access to public goods and services is a sine qua non condition. The elements that make up the scenario of negligence regarding the health needs of Venezuelans in Boa Vista can also be evidenced in the individual dimension of vulnerabilities, in which the obstacles generated by the difference in Portuguese and Spanish languages represent the main vulnerabilities identified among the Venezuelan immigrant population and prevent quality access to health goods and services.

## CONCLUSION

The objective reality of the Venezuelan immigrant residing in Roraima, specifically in the capital Boa Vista, presents multiple vulnerabilities and unmet health needs. In the structural dimension, the mode of production and social reproduction creates elements in the other dimensions that determine the exclusion of Venezuelans in the various sectors of society, even preventing their access to quality health goods and services. Language barriers, xenophobia and work overload make it difficult for Nursing to respond adequately to the health needs of Venezuelans in Boa Vista. The mode of production and social reproduction determines both the vulnerabilities of Venezuelans and the way in which institutions and organizations work to deal with them.

The Venezuelan migratory phenomenon in Roraima is of such magnitude that it requires progress in migration studies, especially those related to Venezuelan indigenous people, such as the Warao, the largest ethnic group that migrates to Brazil. Along with gender, the race/ethnicity category can contribute to a deeper understanding of the health needs and vulnerabilities of Venezuelan immigrants in Brazil.
