# ERRATUM: Vulnerability of Venezuelan immigrants living in Boa Vista, Roraima

**DOI:** 10.1590/1980-220X-REEUSP-2023-0074ener

**Published:** 2023-11-20

In the article “Vulnerability of Venezuelan immigrants living in Boa Vista, Roraima”, with the DOI: https://doi.org/10.1590/1980-220X-REEUSP-2023-0074en, published by the journal “Revista da Escola de Enfermagem da USP, volume 57 of 2023 (spe), on page 1:

**Where was written:**

Corresponding author:

Aristides Sampaio Cavalcante Neto

Rua Manoel Dias de Almeida, 862

69305280 - Boa Vista, RR, Brazil

aristides.neto@alumni.usp.br

**Now read:**

Corresponding author:

Aristides Sampaio Cavalcante Neto

Avenida Glaycon de Paiva, 2426

69303340 – Boa Vista, RR, Brazil

aristides.neto@alumni.usp.br

